# The Effect of Hypoxic and Normoxic Culturing Conditions in Different Breast Cancer 3D Model Systems

**DOI:** 10.3389/fbioe.2021.711977

**Published:** 2021-11-04

**Authors:** Andreas Svanström, Jennifer Rosendahl, Simona Salerno, Emma Jonasson, Joakim Håkansson, Anders Ståhlberg, Göran Landberg

**Affiliations:** ^1^ Department of Laboratory Medicine, Institute of Biomedicine, Sahlgrenska Center for Cancer Research, Sahlgrenska Academy, University of Gothenburg, Gothenburg, Sweden; ^2^ Unit of Biological Function, Division Materials and Production, RISE Research Institutes of Sweden, Borås, Sweden; ^3^ Department of Laboratory Medicine, Institute of Biomedicine, Gothenburg University, Gothenburg, Sweden; ^4^ Wallenberg Centre for Molecular and Translational Medicine, University of Gothenburg, Gothenburg, Sweden; ^5^ Department of Clinical Genetics and Genomics, Region Västra Götaland, Sahlgrenska University Hospital, Gothenburg, Sweden; ^6^ Department of Clinical Pathology, Sahlgrenska University Hospital, Gothenburg, Sweden

**Keywords:** 3D printed scaffolds (3DPS), alginate, patient-derived scaffolds (PDS), breast cancer, normoxia, hypoxia

## Abstract

The field of 3D cell cultures is currently emerging, and material development is essential in striving toward mimicking the microenvironment of a native tissue. By using the response of reporter cells to a 3D environment, a comparison between materials can be assessed, allowing optimization of material composition and microenvironment. Of particular interest, the response can be different in a normoxic and hypoxic culturing conditions, which in turn may alter the conclusion regarding a successful recreation of the microenvironment. This study aimed at determining the role of such environments to the conclusion of a better resembling cell culture model to native tissue. Here, the breast cancer cell line MCF7 was cultured in normoxic and hypoxic conditions on patient-derived scaffolds and compared at mRNA and protein levels to cells cultured on 3D printed scaffolds, Matrigel, and conventional 2D plastics. Specifically, a wide range of mRNA targets (40), identified as being regulated upon hypoxia and traditional markers for cell traits (cancer stem cells, epithelial–mesenchymal transition, pluripotency, proliferation, and differentiation), were used together with a selection of corresponding protein targets. 3D cultured cells were vastly different to 2D cultured cells in gene expression and protein levels on the majority of the selected targets in both normoxic and hypoxic culturing conditions. By comparing Matrigel and 3DPS-cultured cells to cells cultured on patient-derived scffolds, differences were also noted along all categories of mRNA targets while specifically for the GLUT3 protein. Overall, cells cultured on patient-derived scaffolds closely resembled cells cultured on 3D printed scaffolds, contrasting 2D and Matrigel-cultured cells, regardless of a normoxic or hypoxic culturing condition. Thus, these data support the use of either a normoxic or hypoxic culturing condition in assays using native tissues as a blueprint to optimize material composition.

## Introduction

Today, there is a rapid development of materials used for 3D cell cultures, including organoids and 3D printed scaffolds, which are replacing conventional 2D cultures and animal experiments (Katt et al., 2016). A vast number of studies point at different key success factors in the development of 3D printing materials, ranging from a category of technical feasibility of producing the desired product to biocompatibility and the physiological relevance of the created microenvironment (Ngo et al., 2018). Several studies have demonstrated the complexity of a tumor microenvironment and point at the heterogeneity of inhabiting cells, and interaction with the immune system as well as biochemical cues given by endocrine signaling and the extracellular matrix (Baghban et al., 2020). In cancer, the constituents of patient-derived scaffolds and the response of inhabiting reporter cells have been linked to clinical data (Landberg et al., 2020a; Landberg et al., 2020b), underscoring the importance of the extracellular matrix in creating a physiologically relevant microenvironment. Importantly, the success in manufacturing a 3D material with a relevant physiological microenvironment can be analyzed by comparing the response of reporter cells cultured in produced 3D scaffolds to cells cultured in native tissue.

In the tumor microenvironment, cells will experience a gradient of oxygen levels that will influence cell behavior (Al Tameemi et al., 2019; Mas-Bargues et al., 2019). During the development of materials for biological model systems, efforts to improve the material will depend on the cellular response, which in turn may be affected by oxygen levels. This study compares the culturing platforms patient-derived scaffolds (PDS), 3D printed scaffolds (3DPS), Matrigel, and conventional 2D cultures by the response of breast cancer reporter cells in normoxia and hypoxia. The cellular response was assessed by studying gene expression levels using a panel of biomarker genes for the cellular properties of cancer stem cells (CSC), epithelial–mesenchymal transition (EMT), pluripotency, proliferation, and differentiation, and by protein levels using Western blot to identify the best conditions for a relevant tumor cell culture model.

## Methods

### De-Cellularization of Patient-Derived Scaffolds

Patient-derived breast tumors were collected *via* the clinical pathology diagnostic unit at Sahlgrenska University Hospital. Processing of patient material and data has been approved by the Regional Research Ethics Committee in Gothenburg (DNR: 515-12 and T972-18). The tumors were de-cellularized as described in a previous study by Landberg et al. (2020a). In brief, breast tumors were de-cellularized in a lysis buffer containing 0.1% SDS (Sigma-Aldrich), 0.02% sodium azide (VWR), 5 mM 2H_2_O-Na_2_-EDTA (Sigma-Aldrich), and 0.4 mM phenylmethanesulfonyl fluoride (Sigma-Aldrich). Scaffolds were washed in a lysis buffer lacking SDS followed by washing in distilled water and PDS (Medicago). Washed scaffolds were sterilized in 0.1% peracetic acid (Sigma-Aldrich), washed in PBS supplemented with 1% Antibiotic–Antimycotic (ThermoFischer Scientific), and stored at 4°C in PBS (Medicago) containing 0.02% Na-azide (VWR) and 5 mM 2H_2_O-Na_2_-EDTA (Sigma-Aldrich). Prior to use, scaffolds were cut to about 3 × 3 × 3 mm size.

### Bioprinting

Alginate (Protanal LF 10/60, FMC) with hydroxyapatite (Sigma-Aldrich) was prepared and printed as described in a study by Svanström et al. (2021). In brief, 8% (v/v) alginate and 5% (w/v) hydroxyapatite were mixed using an Ultra-Turrax T50 digital dispenser (IKA), and printed in 4 layer discs ⌀20 mm using an EnvisionTEC 4th Gen 3D-Bioplotter^®^ (EnvisionTEC) and a needle diameter of 400 µm. Printed scaffolds were cross-linked during printing with 0.1 M CaCl_2_ (VWR).

### Cell Culture

MCF7 cells (ATCC HTB-22) were kept sub-confluent in DMEM (Thermo Fischer Scientific) supplemented with a final concentration of 10% (v/v) FBS (Sigma-Aldrich), 1% (v/v) penicillin/streptomycin (Sigma-Aldrich), 1% (v/v) MEM non-essential amino acids (Sigma-Aldrich), and 1% (v/v) L-glutamine (Sigma-Aldrich), and cultured at 37°C at 5% CO_2_. Cells were kept in normoxic conditions (21% O_2_) if nothing else started. Cell suspensions were prepared by washing cells in PBS and by detaching cells using trypsin-EDTA (Thermo Fischer Scientific). Detached cells were washed in supplemented media, centrifugated at 300 × *g* for 3 min, and the pellet was resuspended in above-described DMEM.

3DPS and PDS were placed in a 24-well plate (Sarstedt) with supplemented media for 1 h prior to cell seeding. Cells were seeded at a cell density of 300,000 cells/ml in a total volume of 2 ml and cultured at 37°C at 5% CO_2_. Following 24 h of initial culture, 3DPS and PDS were moved every 3–4 days to a new 6-well plate (Sarstedt) until reaching a total culturing time of 3 weeks. 2D cultured cells were seeded at a cell density of 12,500 cells/ml in a total volume of 2 ml in a 6-well plate (Sarstedt) and cultured for 72 h. Cells cultured for 3 weeks or 72 h in 3D or 2D, respectively, were placed in normoxia (21% O_2_) or hypoxia (1% O_2_) at 37°C and 5% CO_2_ for 48 h. A hypoxic environment was achieved using a Sci-tive-N hypoxia chamber (Ruskinn).

### RNA Purification and qPCR

RNA was purified and analyzed by quantitative PCR (qPCR) as described previously (Weigelt et al., 2014). Briefly, scaffolds were rinsed in supplemented media, lysed in QIAzol, and disrupted using a TissueLyser II (Qiagen). RNA was extracted using an automated extraction robot (QIAcube, Qiagen) configured to miRNeasy Micro Kit reagents (Qiagen) with on-column DNA digestion (Qiagen). Nucleic acid concentration was measured using Nanodrop ND-1000 (Saveen Werner). Complementary DNA (cDNA) was produced with a GrandScript cDNA Synthesis Kit (TATAA Biocenter) using 20 µl reactions, and a T100 Thermal Cycler (Bio-Rad) at 22°C for 5 min, 42°C for 30 min, and 85°C for 5 min followed by cooling to 4°C. Samples were diluted 1:4 in RNase-free water (Invitrogen). qPCR was performed on a CFX384 (Bio-Rad) in 6 µl reactions, containing 400 nM of each primer (Supplementary Table S1), 1x SYBR Grandmaster (TATAA Biocenter), and 2 µl diluted cDNA. The temperature profile was as follows: 95°C for 2 min, 39 cycles of amplification at 95°C for 5 s, 60°C for 20 s, and 70 °C for 20 s followed by a melting curve analysis at 65°C–95°C with 0.5°C per 5 s increments. Cycles of quantification (Cq) values by the regression method were determined using CFX Manager software version 3.1 (Bio-Rad) and analyzed using GenEx (MultiD). Missing values were imputed based on replicates followed by setting the remaining missing values to +1 of the group. Values higher than Cq-values of 35 were set to 35. Values were normalized by reference genes evaluated by the NormFinder algorithm and transformed to relative values and log2 scale. Analysis and the t-distributed stochastic neighbor embedding (t-SNE) plot were performed in MATLAB (Mathworks).

### Western Blot

Cells cultured in 2D were washed once in PBS (VWR) and detached using trypsin–EDTA (ThermoFischer Scientific). Cells cultured on 3DPS and PDS were transferred to a 24-well plate, gently washed twice in supplemented media, and detached using trypsin–EDTA (Thermo Fischer Scientific) in an Incu-shaker (Benchmark) for 5 min at 130 rpm followed by manual pipetting. Cells cultured on Matrigel were detached as 3DPS and PDS-cultured cells, where a Matrigel/cell suspension was made during the manual pipetting step. All detached cells were washed in supplemented media, centrifugated at 300 × *g* for 3 min, and resuspended in PBS (VWR). Cells were filtered through a 35 µm mesh (Corning), washed in PBS (VWR), centrifugated at 300 × g for 3 min, and resuspended in PBS (VWR). The washing step was repeated twice. Cell pellets were lysed in a RIPA buffer containing 1x Halt protease and phosphatase inhibitors with EDTA (all ThermoFischer Scientific). Lysates were centrifuged at 14,000 × g for 15 min at 4°C and the supernatant was recovered and stored at −20°C prior to analysis. Protein levels were estimated using the DC protein assay (Bio-Rad) with BSA standard (Thermo scientific). All samples were denatured under reducing conditions at 98°C for 10 min and analyzed together with a pre-stained protein ladder (Thermo Scientific) on a 20% SDS-PAGE gel (Bio-Rad). Proteins were transferred to a nitrocellulose membrane (GE Healthcare) and stained with SYPRO RUBY (Invitrogen), according to the manufacturer’s instructions. Membranes were imaged/analyzed for total protein content using a Gel doc and Image Lab software (Bio-Rad). Following total protein staining, membranes were blocked in a blocking buffer for 30 min at room temperature, incubated with primary antibodies for 1 h at room temperature in the blocking buffer, washed in PBS (VWR) with 0.1% Tween 20 (Sigma-Aldrich), and incubated with secondary HRP-conjugated antibodies in the blocking buffer for 1 h at room temperature Membranes were washed in a washing buffer and incubated with ECL select (GE healthcare). Chemiluminescence was detected using ImageQuant800 (Amersham), and images were analyzed using ImageJ ((Schneider et al., 2012); see Supplementary Table S2 for antibody specifications). The signals given by the specific antibodies from a blot containing all samples per biological replicate were first normalized to the total loading per sample as measured by SYPRO RUBY staining and then to the total signal over all samples.

### Data Analysis

**FIGURE 1:**
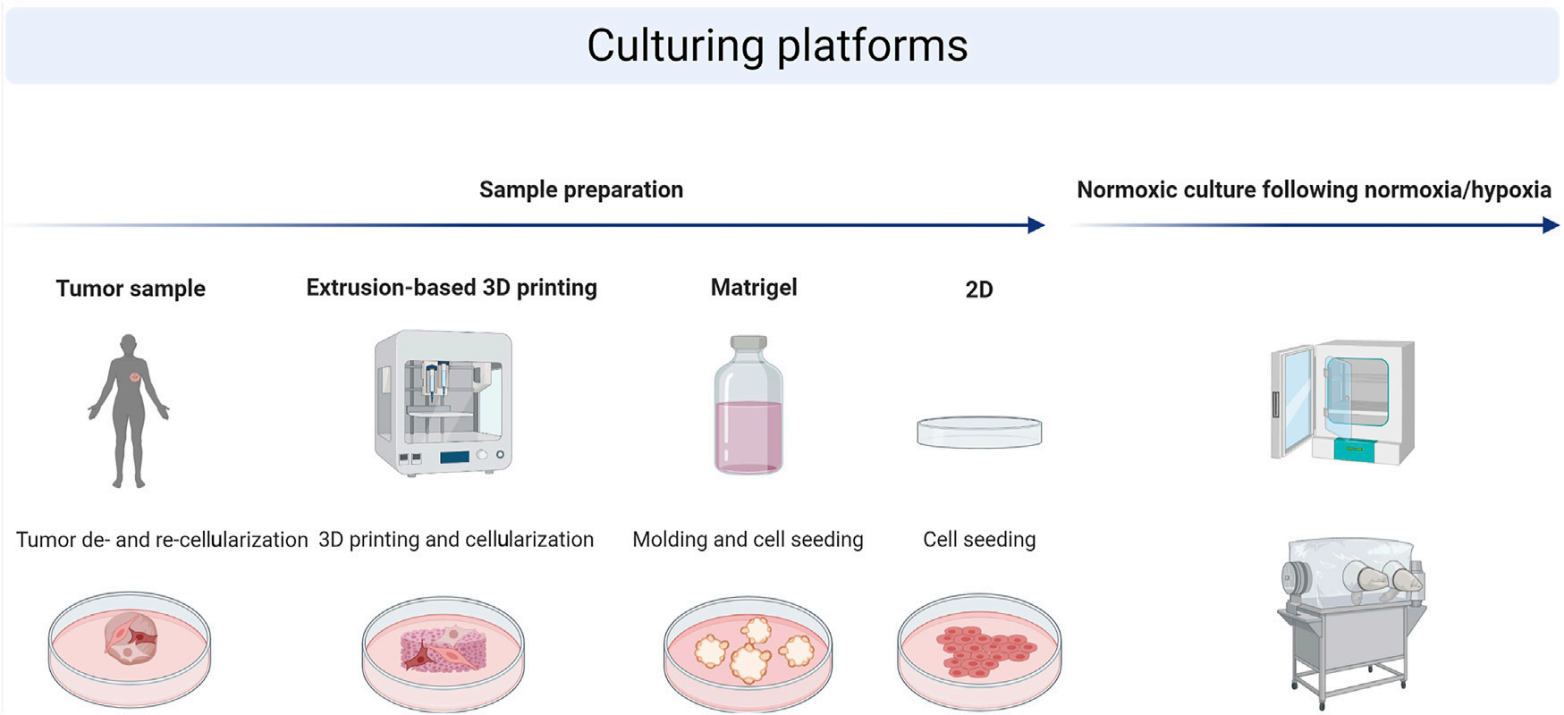
Schematic illustration of the experimental workflow. The following platforms were used for cell culture on patient-derived scaffolds (PDS), 3D printed scaffolds (3DPS), molded Matrigel, or in 2D culture plates. Cells were seeded on top of each platform, cultured for 3 weeks (3D) or 72 h (2D) followed by a 48 h culture in either normoxia or hypoxia, and analyzed on gene expression and protein levels using qPCR or Western blot, respectively.

All data were analyzed using GraphPad Prism v8 (GraphPad). Illustration in Figure 1 was designed with biorender.com.

## Result

The development and optimization of biocompatible materials for 3D printing requires studies on cellular behavior, where the cellular response to the 3D printed microenvironment is compared to an *in vivo*–like system. Depending on the type of research conducted, cells are either cultured in normoxic or hypoxic conditions. It is therefore important to determine if similarities between cells cultured in a 3D printed and an *in vivo*–like environment change between normoxia and hypoxia as this will influence the optimization of 3D printing materials. Here, the breast cancer cell line MCF7 was cultured in 2D culture plates, on 3DPS, Matrigel, or in an *in vivo*–like setting represented by PDS, and the expression levels of genes and proteins known to be regulated by hypoxia were compared (Bando et al., 2003) (Figure 1).

### 3DPS- and PDS-Cultured Cells Display Similar Gene Expression Profiles

**FIGURE 2:**
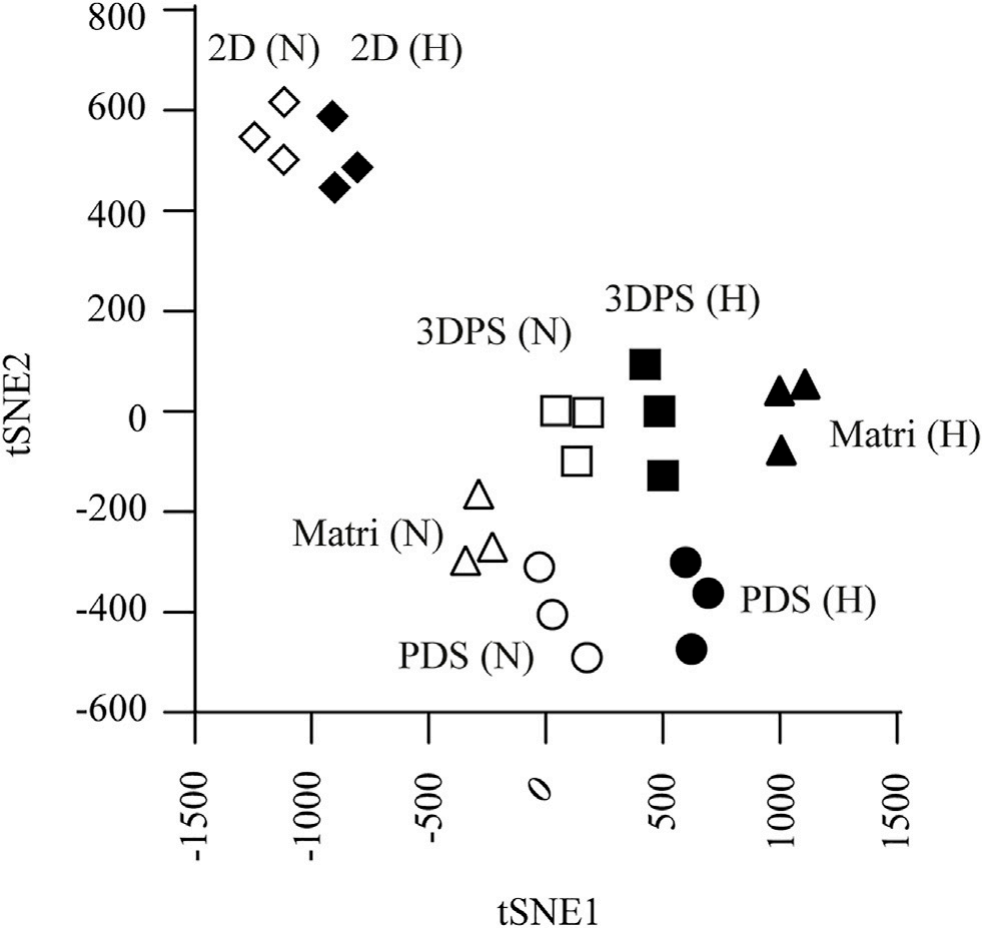
Cell culture systems evaluated by t-SNE analysis. Cells were cultured in normoxia (N, hollow symbols) or hypoxia (H, solid symbols) in patient-derived scaffolds (PDS, circles), 3D printed scaffolds (3DPS, squares), Matrigel (M.Gel, triangles), or 2D (diamonds). Each dot indicates an individual experiment including 3 replicates each.

**FIGURE 3:**
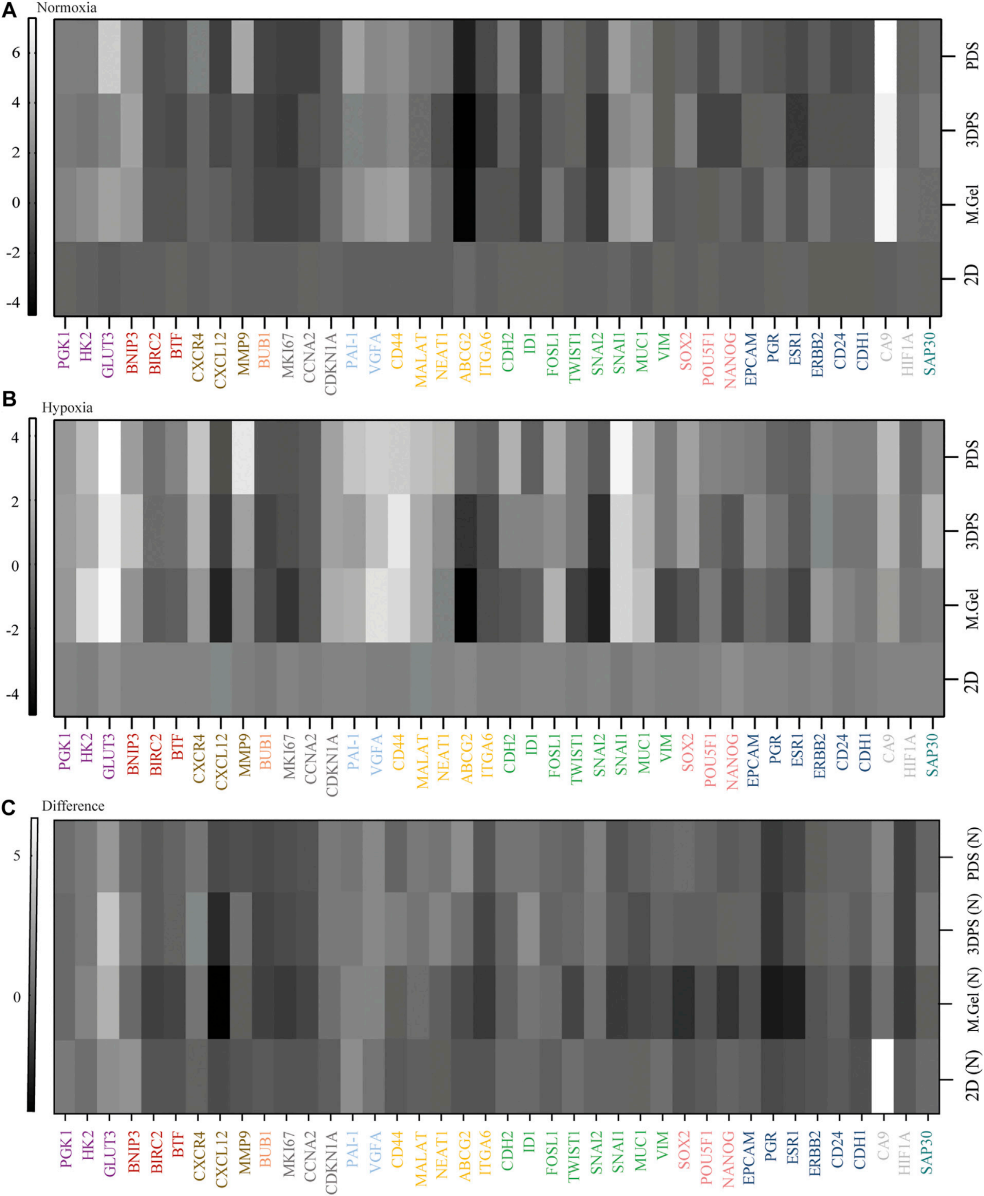
Heat maps representing relative gene expression levels. **(A, B)** Gene expression levels of cells cultured in normoxia **(A)** or hypoxia **(B)** in patient-derived scaffolds (PDS), 3D printed scaffolds (3DPS), and Matrigel (M.Gel) relative to 2D (*n* = 3). **(C)** Difference in gene expression levels between hypoxia and normoxia. Color codes: metabolism (purple), cell death (red), invasion (brown), cell division (orange), proliferation (dark gray), angiogenesis (light blue), cancer stemness (yellow), epithelial–mesenchymal transition (green), pluripotency (pink), differentiation (dark blue), hypoxia (gray), and epigenetics (dark green).

By studying the expression levels of genes representing markers for metabolism, cell death, invasion, cell division, mitosis/proliferation, angiogenesis, cancer stemness, EMT, pluripotency, differentiation, hypoxia, and epigenetics, we could compare the expression profiles of the cells in different environments (Supplementary Table S3). The t-SNE analysis clearly separated samples in normoxic from hypoxic culturing condition as well as by the type of the culturing model (Figure 2). Specifically, 3D cultured cells were shown to have a higher expression of genes related to metabolism (*PGK1*, *HK2*, and *GLUT3*), angiogenesis (*PAI1* and *VEGFA*), cancer stemness (*CD44* and *MALAT1*), invasion (*CXCR4*), epithelial–mesenchymal transition (EMT) (*FOSL1*, *SNAI1*, and *MUC1*), and hypoxia (*CA9*) while having a lower expression of genes related to mitosis/proliferation (*BUB1*, *MKI67*, and *CCNA2*) and differentiation (*ESR*) in normoxic and/or hypoxic culture conditions relative to 2D cultured cells (Figures 3A,B). Noteworthy, 2D cultured cells displayed higher response to hypoxia shown by the hypoxia marker (*CA9*) relative 3D cultured cells (Figure 3C; Supplementary Table S4).

The analysis of gene expression levels in PDS-, 3DPS-, and Matrigel-cultured cells showed more similarities between PDS- and 3DPS-cultured cells than between PDS and Matrigel in normoxia (Figures 3A,B; Supplementary Table S3). In addition, the gene expression response to hypoxia showed a significant difference between Matrigel-cultured cells and 3DPS, PDS, and 2D cultured cells (Figure 3C) (Supplementary Table S5). The decrease in total RNA levels upon hypoxia was shown to be similar between 2D and 3D cultured cells (Supplementary Figure S1), suggesting a similar decrease in viability and/or proliferation between the culture platforms.

### PDS- and 3DPS-Cultured Cells Showed a High Degree of Similarity at the Protein Level

**FIGURE 4:**
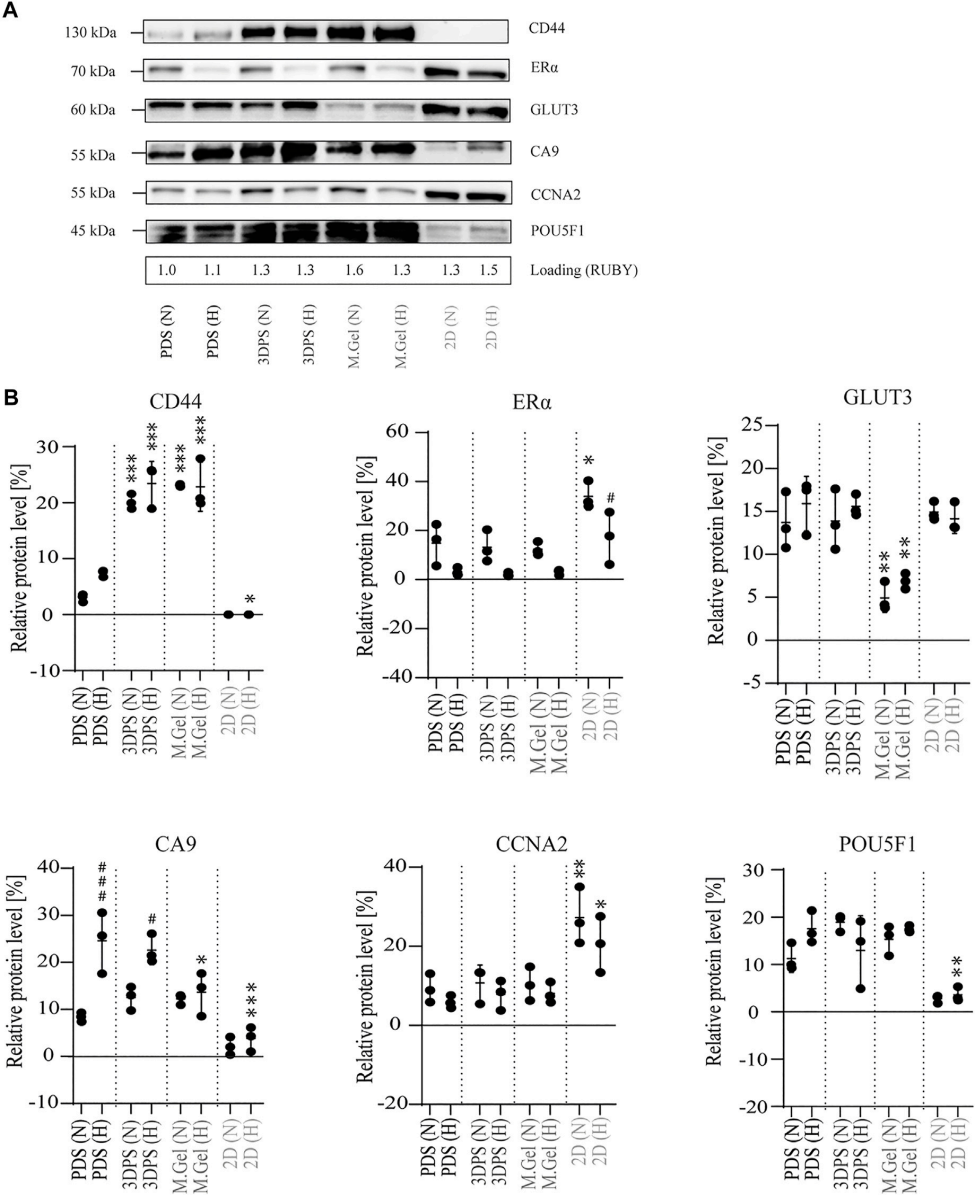
Protein levels in MCF7 cells cultured in normoxia and hypoxia. **(A)** Representative Western blot image from total protein extracts of cells cultured in normoxia (N) or hypoxia (H) on patient-derived scaffolds (PDS), 3D printed scaffolds (3DPS), Matrigel (M.Gel), or in 2D. Protein ladder 45–130 kilodalton (kDa). Loading control by SYBRO Ruby staining (RUBY). **(B)** Quantification of Western blot (*n* = 3) showing the protein amount relative total protein amount. Asterix (*) indicates statistical comparison of respective group and PDS. The hash sign (#) indicates statistical comparison of normoxia and hypoxia for respective platform. The statistical method used was two-way ANOVA; Tukey’s *post hoc* test for multiple comparison; *^,#^ *p*-value <0.05, ** *p*-value <0.01, ***^,###^ *p*-value <0.001.

To further assess the response of MCF7 cells to different culturing platforms, protein analysis using Western blots was performed. 2D cultured cells were shown to have significantly lower protein levels related to stemness (CD44), hypoxia (CA9), and pluripotency (POU5F1) while having higher levels of proteins related to proliferation (CCNA2) and differentiation (ERα) compared to PDS-cultured cells (Figure 4), supporting the gene expression data (Figures 3A,B). Surprisingly, CD44 protein levels were shown to be upregulated in 3DPS- and Matrigel-cultured cells relative to PDS-cultured cells, contrasting gene expression levels. In addition, the protein levels of the glucose transporter GLUT3 was significantly downregulated in Matrigel-cultured cells relative to PDS-cultured cells. However, no difference in the uptake of a glucose analog among PDS-, 3DPS-, and Matrigel-cultured cells was shown (Supplementary Figure S2).

By comparing the response to hypoxia, PDS- and 3DPS-cultured cells showed significant upregulation of CA9, while Matrigel- or 2D-cultured cells showed no difference (Figure 4B), consistent with gene expression. The decrease in the total protein level upon hypoxia was significantly lower in 2D than in PDS-cultured cells (Supplementary Figure S1).

## Discussion

Several methods may be used to compare the cellular response to a microenvironment, including prote-, lipid-, secret-, and transcriptomics among others that are time consuming and labor intensive while generating large and unbiased datasets. This study uses qPCR as a simple and fast, yet highly sensitive, method to detect minor changes in cell response to the microenvironment by analyzing markers important for cancer characteristics (CSC, EMT, pluripotency, proliferation, and differentiation) and known to be affected by hypoxia and to describe main cellular traits. Instead of using primary tumor cells, which carry different characteristics from patient to patient, we used the standardized cancer cell line MCF7 to be able to analyze the effect of the different microenvironments on the cells. Here, the comparison among the culturing platforms such as PDS, 3DPS, Matrigel, and 2D in normoxia and hypoxia has shown major differences between 2D and 3D. 3D cultured cells were indicated by a gene expression level analysis to have higher levels of genes related to metabolism (*PGK1*, *HK2*, and *GLUT3*). These results are consistent with previous studies, where *GLUT3* expression levels were regulated by hypoxic conditions (Navale and Paranjape, 2016), here indicated by a relatively high level of a hypoxia marker (*CA9*) and markers for angiogenesis (*PAI1* and *VEGFA*) in both normoxic and hypoxic culturing conditions. As cells cultured on 3DPS do not infiltrate the material and are suggested to grow in layers less than 1–200 µm thick, a distance required to reach hypoxia (Carmeliet and Jain, 2000), the relative hypoxic conditions during normoxic culture may instead be due to cell density (Wenger et al., 2015). 3D cultured cells were also shown to have lower levels of genes related to cell proliferation or mitosis (*CCNA2*, *MKI67*, and *BUB1*) and differentiation (*ESR1*) relative 2D cultured, consistent with higher levels of a cell cycle inhibitor (*CDKN1*) and cancer stemness–related markers (*CD44* and *MALAT1*). Noteworthy, the expression of *ITGA6*, an integrin important for mammosphere formation and associated with stemness traits (Cariati et al., 2008), was downregulated in a 3D setting compared to 2D. This may be explained by differences in cell polarity, where integrins are required for maintaining cell polarity (Lee and Streuli, 2014) and where 2D cultured cells are known to be highly polar in comparison to 3D cultured cells (Baker and Chen, 2012). In addition, ABCG2, a marker for cancer stemness that is related to cancer drug resistence and shown to be upregulated upon doxorubucin treatment in a 3D environment, (Svanström et al., 2021) and was downregulated in a 3D environment. *ABCG2* expression is controlled by several different transcriptional factors, including *ESR1* (Mo and Zhang, 2012) which here was downregulated on the protein level in 3D relative 2D cultured cells, supporting a downregulation of *ABCG2*. 3D cultured cells were also shown to have an increased matrix remodeling profile relative 2D cultured cells by increased levels of a matrix degrading protease (*MMP9*), a chemotaxis receptor (*CXCR4*), as well as reduced levels of the CXCR4-related chemokine (*CXCL12*) whose downregulation is suggested to promote breast cancer metastasis (Yu et al., 2017).

Interestingly, 3D cultured cells showed an increased differential expression of EMT-related genes with low levels of *SNAI2* and high levels of *SNAI1* relative 2D cultured cells. Although both *SNAI1* and *SNAI2* are correlated with EMT by suppressing the expression of E-cadherin (*CDH1*) (Serrano-Gomez et al., 2016), a recent study showed that a high ratio of *SNAI2/SNAI1* increased the levels of the mTOR signaling protein PDL2 that regulates cell growth and proliferation (Ganesan et al., 2016). Thus, the reduced *SNAI2/SNAI1* ratio correlates with the decreased levels of proliferation markers and indicates an upregulated EMT response by *SNAI1*, supported by an upregulation of *FOSL1* and *MUC1* levels. Surprisingly, *ID1* level that is shown by the previous study to be implicated in breast cancer metastasis (Gupta et al., 2007; Gumireddy et al., 2014) was noted to be downregulated. A previous study has shown *ID1* to inhibit *TWIST*-mediated EMT to promote mesenchymal–epithelial transition at metastatic sites where *SNAI1* expression is low and underscores that *ID1* does not affect *SNAI1*-mediated EMT at the primary tumor site (Stankic et al., 2013). In addition, *ID1* was shown to keep cancer stemness with epithelial traits. Thus, the reduction of *ID1*, in an environment with high levels of *SNAI1*, suggests that induced stemness is of less epithelial character. Together, data on the response of the reporter cells to a 3D environment by mRNA expression levels are consistent with those of previous studies and support their use in comparing different 3D models.

By comparing the 3D culturing platforms, 3DPS- and Matrigel-cultured cells were shown to have significant differential gene expression levels in 9 and 12 out of 40 genes, respectively, in normoxia compared to PDS-cultured cells, which increased to 15 genes for both models, in hypoxia. Thus, 3DPS- and Matrigel-cultured cells are more similar to PDS-cultured cells in a normoxic environment than hypoxic environment, although the directional response in gene expression relative 2D cultured cells was similar in both culturing conditions. In addition, PDS-cultured cells were more similar to 3DPS-cultured cells than Matrigel-cultured cells. This was supported by the response in gene expression levels to hypoxia, where Matrigel-cultured cells were different from PDS-, 3DPS-, and 2D cultured cells. The relative similar response to hypoxia between 2D and 3D (PDS, 3DPS), as analyzed by response in gene expression levels in the defined set of markers, was surprisingly given difference in overall cell response between 2D and 3D. However, 2D cultured cells had a relatively high response to hypoxia compared to 3D cultured cells, as measured by the hypoxia marker (*CA9*), indicating that 2D cultured cells respond differently to hypoxia. Thus, the lack of larger differences in response between 2D and 3D cultured cells upon hypoxia may be due to the selection of genes included in the gene panel.

To verify the findings on gene expression levels, Western blot was performed on selected targets, where protein loadings were normalized to total protein levels (Supplementary Figure S3) as hypoxia affected common loading controls (actin, tubulin) (Supplementary Figure S4). Consistent with the gene expression in this study, and protein levels within a previous study (Landberg et al., 2020a), 3D cultured cells had upregulated protein levels of markers for cancer stemness (CD44) and pluripotency (POU5F1), and downregulated levels of markers for proliferation (CCNA2) and differentiation (ERS1). Surprisingly, GLUT3 levels were similar among 2D-, PDS-, and 3DPS-cultured cells, while Matrigel-cultured cells showed relatively low protein levels of GLUT3, contrasting data on gene expression levels. Thus, GLUT3 homeostasis is indicated to be different in 2D and Matrigel-cultured cells relative PDS- and 3PDS-cultured cells. This may also be the case for CD44, whose protein levels among PDS, 3DPS, and Matrigel did not correspond to the gene expression. In addition, data from a glucose intake assay showed similar uptake of a glucose analog between 3D cultured cells and a relatively high intake in 2D cultured cells. As glucose uptake is mediated *via* several GLUT transporters (Navale and Paranjape, 2016), the discrepancy between the protein level and glucose intake may be due to a differential control of glucose uptake.

## Conclusion

A comparison among PDS-, 3DPS-, Matrigel-, and 2D cultured cells was performed by studying the gene and protein expression levels of selected genes, where 2D cultured cells separated from 3D cultured cells and where 3DPS- and PDS-cultured cells showed a higher degree of similarity than Matrigel cultured cells. The gene expression and protein levels in PDS- and 3DPS-cultured cells were similar in both normoxia and hypoxia. Thus, conclusion made from assays used to analyze the progress in material development toward an *in vivo*–like environment, here by the cellular response in reporter cells cultured in a 3D printed material, is indicated to be equally valid in a normoxic and hypoxic environment.

## Data Availability

The original contributions presented in the study are included in the article/Supplementary Material; further inquiries can be directed to the corresponding author.
