# Supplementary figures and images for: The Effect of Hypoxic and Normoxic Culturing Conditions in Different Breast Cancer 3D Model Systems
Source: Front Bioeng Biotechnol. 2021 Nov 4;9:711977. doi: 10.3389/fbioe.2021.711977 (PMC8632655; doi:10.3389/fbioe.2021.711977)

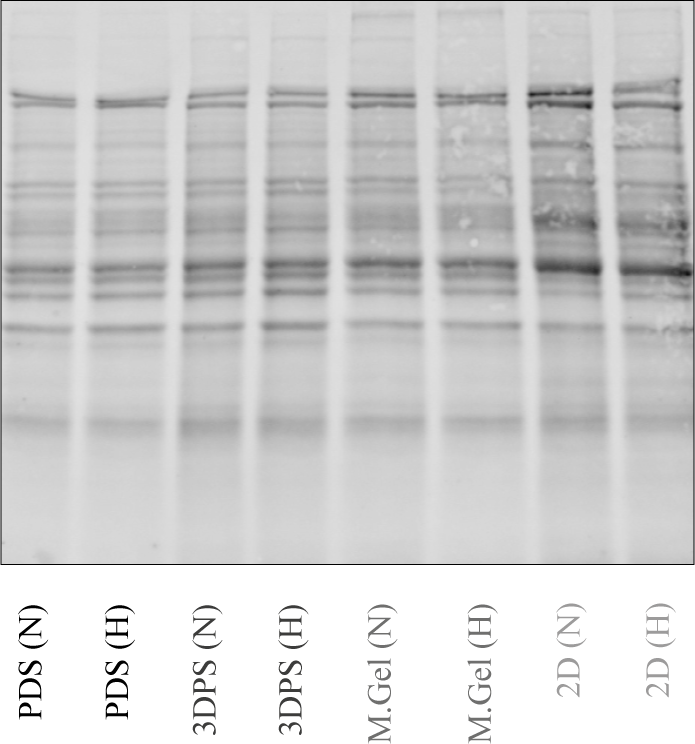

Supplement: Supplementary file 1 [file Image3.TIF]

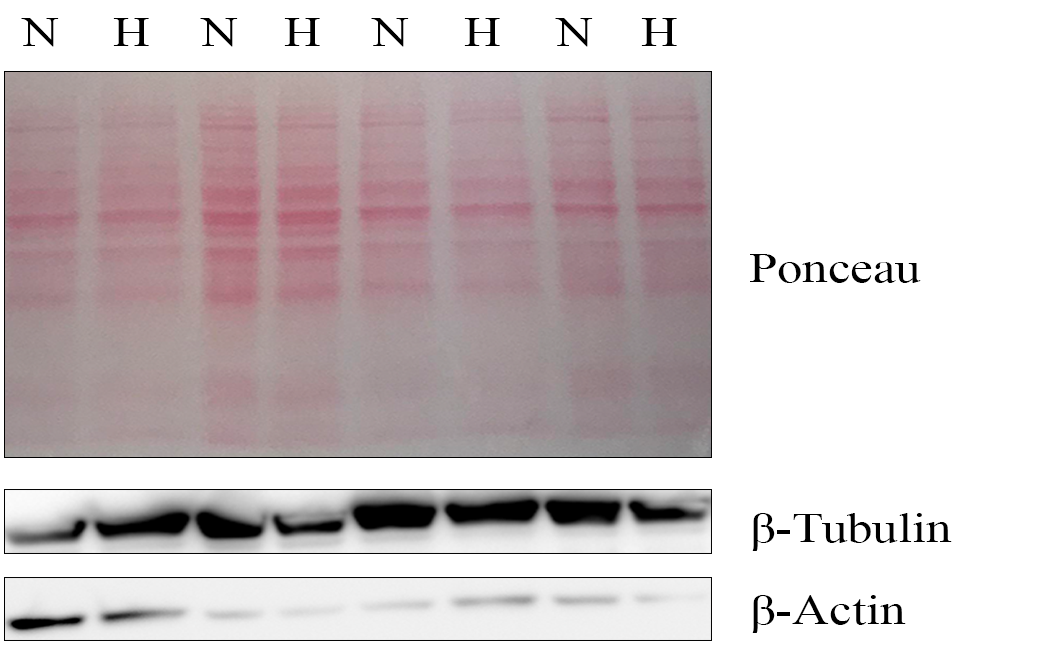

Supplement: Supplementary file 2 [file Image4.TIF]

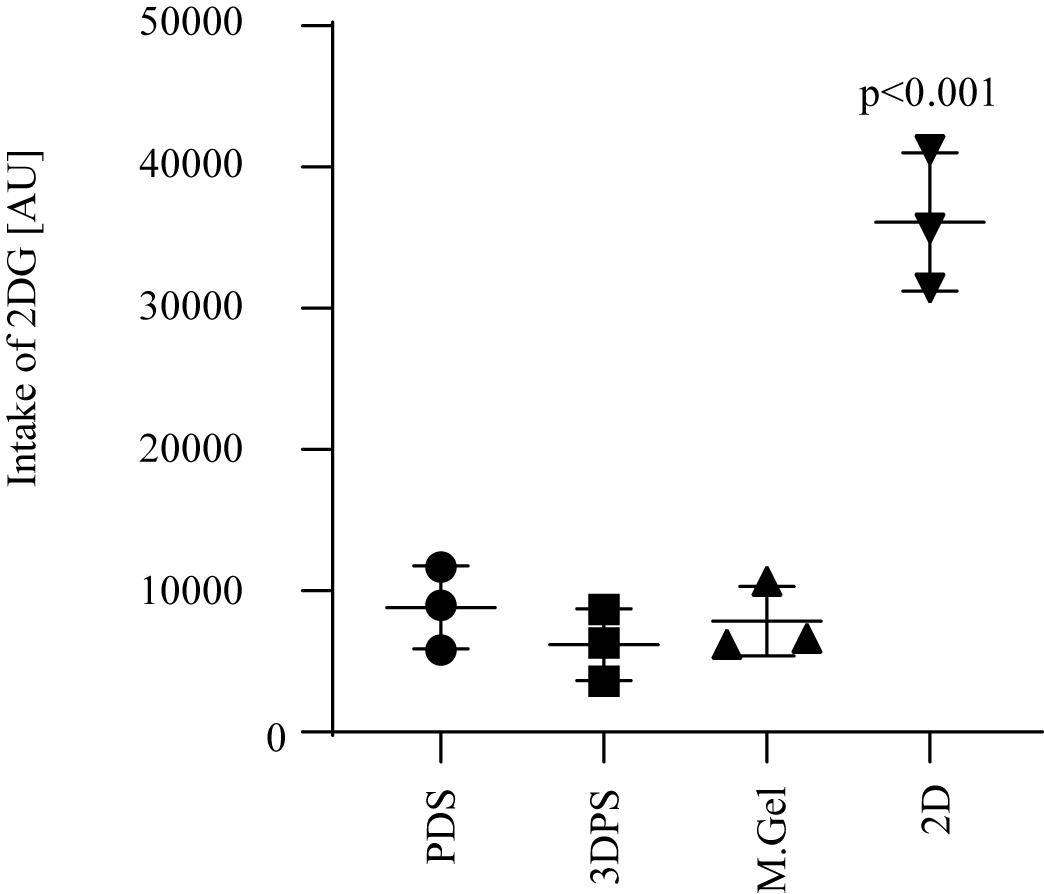

Supplement: Supplementary file 3 [file Image2.TIF]

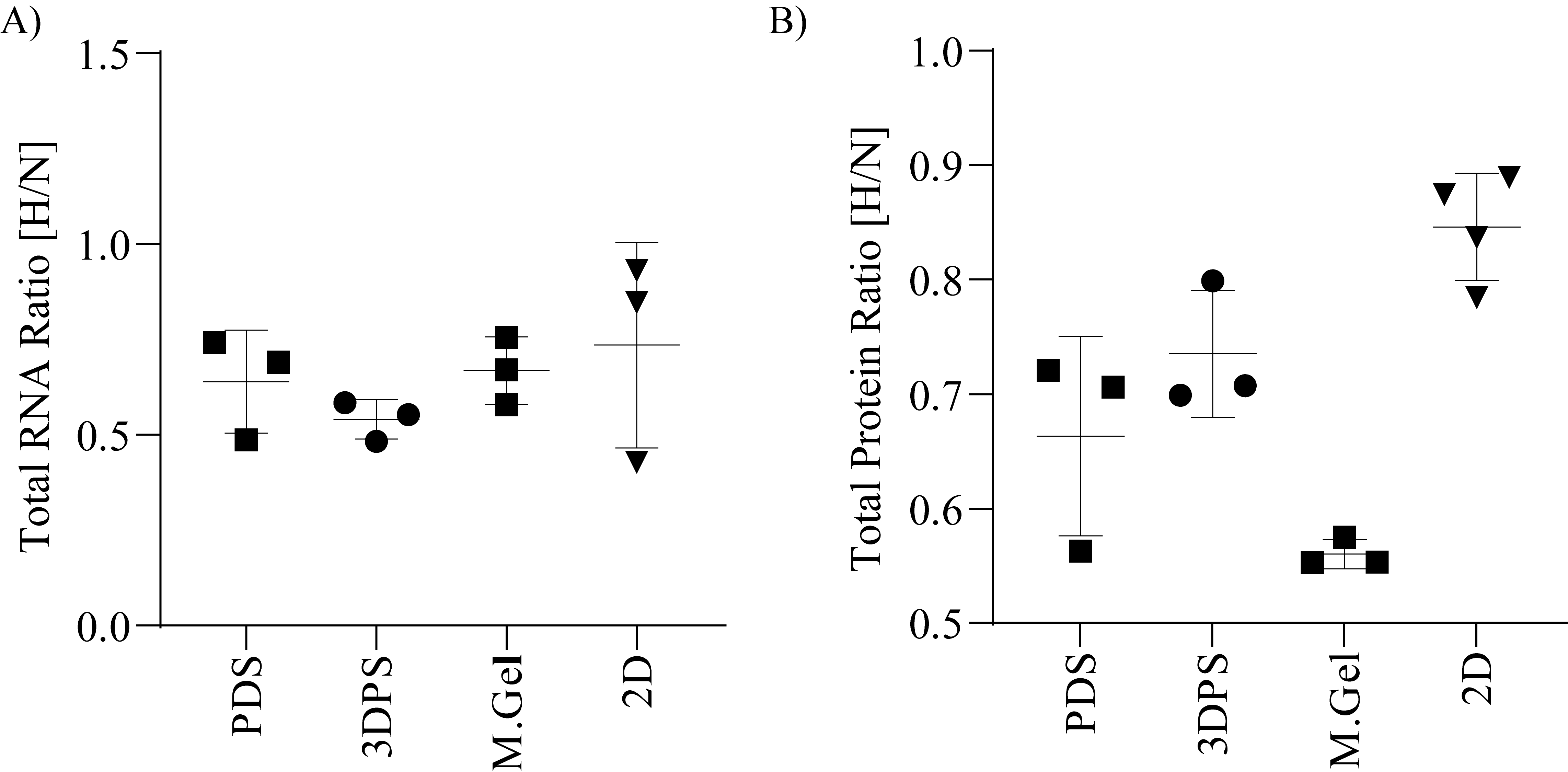

Supplement: Supplementary file 4 [file Image1.TIF]
