# Supplementary material for: The Effect of Hypoxic and Normoxic Culturing Conditions in Different Breast Cancer 3D Model Systems
Source: Front Bioeng Biotechnol. 2021 Nov 4;9:711977. doi: 10.3389/fbioe.2021.711977 (PMC8632655; doi:10.3389/fbioe.2021.711977)
Supplement: Supplementary file 6 [file DataSheet1.docx]

Supplementary Material

# Supplementary Data

## Figures

S Figure 1. Ratio of RNA and protein levels (Hypoxia/Normoxia). Cells cultured in normoxia (N) or hypoxia (H) on patient derived scaffolds (PDS), 3D printed scaffolds (3DPS), Matrigel (M.Gel), or in 2D. (A) RNA levels (n=3). (B) Protein levels (n=3-4). P-value of comparison between PDS and 2D. One-way ANOVA; Tukey’s post-hoc test for multiple comparison.

S Figure 2. Glucose uptake. Cells cultured in normoxia (N) or hypoxia (H) in patient derived scaffolds (PDS), 3D printed scaffolds (3DPS), Matrigel (M.Gel), or 2D were evaluated by their ability to increase intracellular levels of the glucose analogue 2DG (n=3). P-value of a comparison between PDS and 2D. One-way ANOVA; Tukey’s post-hoc test for multiple comparison.

S Figure 3. Loading control using SYRBO Ruby stain. Representative blot of the total protein level used to normalize western blot signals.

S Figure 4. Loading controls and total protein staining. Total cell lysates of cells cultured in normoxia and hypoxia were separated by SDS-PAGE, stained for total protein levels by ponceau staining and probed for actin and tubulin using standard western blot protocol.

## Tables

S Table 1. Primer sequences used for qPCR.

|  | | Gene name | | Forward primer | Reverse primer | |  |
| --- | --- | --- | --- | --- | --- | --- | --- |
| Metabolism | *PGK1* | | TGACCGAATCACCGACCTCT | | | AAGGACTACCGACTTGGCTC | |
|  | *HK2* | | CCCCTGCCACCAGACTAAACTA | | | ACAACGTCTCTGCCTTCCACT | |
|  | *GLUT3* | | ACATTTTGAAGGTTTTGTTGGCTG | | | GGCATTTCCCTTGTCCGTC | |
| Cell death | *BNIP3* | | TCCATCTCTGCTGCTCTCTCAT | | | AGAAGTAATCCACTAACGAACCAAG | |
|  | *BIRC2* | | ACGTCATCGTGCGTCAGA | | | ACGTCATCGTGCGTCAGA | |
|  | *BTF* | | CATCCTCTTCAGCATCACCTTCT | | | TGTCCCAGCAAAAACTCCTCT | |
| Invasion | *CXCR4* | | CCGACCTCCTCTTTGTCATC | | | ACCACCTTTTCAGCCAACAG | |
|  | *CXCL12* | | CAAGGTCGTGGTCGTGCT | | | GCACAGTTTGGAGTGTTGAGA | |
|  | *MMP9* | | CAGTCCACCCTTGTGCTCTT | | | CGACTCTCCACGCATCTCT | |
| Cell division | *BUB1* | | TCACTTGGGACTGTTGATGCT | | | CTTGGGCTTGATGGCTGGAA | |
| Proliferation | *CCNA2* | | AAGACGAGACGGGTTGC | | | GGCTGTTTACTGTTTGCTTTCC | |
|  | *CDKN1* | | TTAGCAGCGGAACAAGGAGT | | | TTAGCAGCGGAACAAGGAGT | |
|  | *MKI67* | | TGGGTCTGTTATTGATGAGCC | | | CATCAGGGTCAGAAGAGAAGC | |
| Angiogenesis | *PAI1* | | TCTCTGCCCTCACCAACATTC | | | CGGTCATTCCCAGGTTCTCTA | |
|  | *VEGFA* | | GAGGAGGGCAGAATCATCACG | | | ATCGCATCAGGGGCACACAG | |
| CSC | *ABCG2* | | GGTGGAGGCAAATCTTCGTTA | | | AGTGCCCATCACAACATCA | |
|  | *ITA6* | | CTGTGCTTGCTCTACCTGTCG | | | CTCCCGTTCTGTTGGCTCTC | |
|  | *CD44* | | GAAGAAGGTGTGGGCAGAAGA | | | ACCATTTCCTGAGACTTGCTG | |
|  | *NEAT1* | | GCCTTCTTGTGCGTTTCTCG | | | CCCTCCCAGCGTTTAGC | |
|  | *MALAT1* | | CGACGAGTTGTGCTGCTATC | | | TCCTCCAAACCCCAAGACCA | |
| EMT | *CDH2* | | CATTATCAACCCCATCTCGG | | | ACTGTCCCATTCCAAACCTG | |
|  | *ID1* | | CTGAGGGAGAACAAGACCGAT | | | CCCCCTAAAGTCTCTGGTGA | |
|  | *FOSL* | | GCAGGCGGAGACTGACAA | | | GGGGAAAGGGAGATACAAGG | |
|  | *VIM* | | CAGATGCGTGAAATGGAAGA | | | TGGAAGAGGCAGAGAAATCC | |
|  | *SNAI1* | | TAATCCAGAGTTTACCTTCCAGCA | | | AGCCTTTCCCACTGTCCTCA | |
|  | *SNAI2* | | GCCAAACTACAGCGAACTGG | | | AGGAGGTGTCAGATGGAGGA | |
|  | *TWIST1* | | GGACAGTGATTCCCAGACGG | | | CATAGTGATGCCTTTCCTTTCAG | |
|  | *MUC1* | | CTGGTCTGTGTTCTGGTTGC | | | CCACTGCTGGGTTTGTGTAA | |
| Pluripotency | *SOX2* | | ACACCAATCCCATCCACACT | | | CCTCCCCAGGTTTTCTCTGT | |
|  | *NANOG* | | CCTATGCCTGTGATTTGTGG | | | AAGTGGGTTGTTTGCCTTTG | |
|  | *POU5F1* | | CGAAAGAGAAAGCGAACCAG | | | AACCACACTCGGACCACATC | |
| Differentiation | *PGR* | | TAAATGAACAGCGGATGAAAGAA | | | CGACACAACTCCTTTTTGCCT | |
|  | *ESR1* | | TGACTATGCTTCAGGCTACCAT | | | ACCTTTCATCATTCCCACTTC | |
|  | *ERBB2* | | ACCTGGAACTCACCTACCTG | | | TCACTTGGTTGTGAGCGATG | |
|  | *CD24* | | GCTCCTACCCACGCAGATT | | | GGTGGTGGCATTAGTTGGAT | |
|  | *CDH1* | | AGAGGACCAGGACTTTGACTTG | | | CAGAGAATCATAAGGCGGGG | |
|  | *EPCAM1* | | CAGGAAGAATGTGTCTGTGAAAACT | | | TTCATTTCTGCCTTCATCACC | |
| Hypoxia | *CA9* | | AGAAATCGCTGAGGAAGGCTC | | | CAGGGCGGTGTAGTCAGAGA | |
|  | *HIFA* | | CGATTTTGGCAGCAACGACACA | | | CGTTTCAGCGGTGGGTAATGGA | |
| Epigenetics | *SAP30* | | GAGCGCAAGGCATCTTTACA | | | GTGGCAACCAACTATCTCAACA | |
| Reference | *GAPDH* | | AGTCAGCCGCATCTTCTTTT | | | CGCCCAATACGACCAAAT | |
|  | *RPS10* | | AGCCGCAGAGATGTTGATG | | | CCTCGGGACTTGAGAGACTG | |
|  | *RPS26* | | GATGCGTGCCCAAGGAC | | | CAGGTCTAAATCGGGGTGG | |
|  | *YWHA* | | ACGCCTCACTCCCGTTT | | | CTGGATGTTCTGCTGGCTC | |

S Table 2. Antibody specifications

| **Antibody** | **ID#** | **Company** | **Dilution** | **Blocking buffer** |
| --- | --- | --- | --- | --- |
| **CA9** | ab15086 | Abcam | 1:1000 | *5% (w/v) low fat milk (Merck) in PBS (VWR)* |
| **CD44** | ab16728 | Abcam | 1:250 |  |
| **CCNA2** | ab181591 | Abcam | 1:20.000 |  |
| **β-TUBULIN** | ab6046 | Abcam | 1:20.000 |  |
| **GLUT3** | ab191071 | Abcam, | 1:1000 | *5% (w/v) low fat milk (Merck) in PBS with 0.2% (v/v) of Tween20 (Sigma-Aldrich)* |
| **POU5F1** | H00005460-M05 | Abnova | 1:1000 |  |
| **ER**α | M7047 | DAKO | 1:1000 | *5% (w/v) BSA (Thermo scientific) in PBS (VWR) with 0.1% (v/v) Tween20 (Sigma-Aldrich)* |
| **ACTIN** | SC47778 | Santa Cruz | 1:1000 | *5% (w/v) low fat milk (Merck) in PBS with 0.1% (v/v) of Tween20 (Sigma-Aldrich)* |
| **Anti-mouse**  **(Secondary)** | HAF007 | R&D systems | 1:2.000 | *Same as primary antibody* |
| **Anti-rabbit**  **(Secondary)** | HAF008 | R&D systems | 1:2.000 | *Same as primary antibody* |

S Table 3. Statistical analysis of differences in gene expression levels of certain genes in response to hypoxia. Data is represented by average log 2 ± SEM.

Two-way ANOVA, Tukey’s post-hoc test for multiple comparison; n.s. = non-significant. Bold/Italic indicates significantly regulated compared to normoxia/hypoxia.

**S Table 4**. Statistical analysis of differences in gene expression levels of certain genes in normoxic and hypoxic culturing conditions compared to 2D cultured cells. Data is represented by average log 2 ± SEM.

Two-way ANOVA, Tukey’s post-hoc test for multiple comparison; n.s. = non-significant. Bold/Italic indicates significantly regulated compared to 2D samples.

S Table 5. Statistical analysis of differences in gene expression levels of certain genes in response to hypoxia relative PDS cultured cells. Data is represented by average log 2 ± SEM.

Two-way ANOVA, Tukey’s post-hoc test for multiple comparison; n.s. = non-significant. Bold/Italic indicates significantly regulated compared to PDS samples in hypoxia.
